# Binary Transformer Based on the Alignment and Correction of Distribution

**DOI:** 10.3390/s24248190

**Published:** 2024-12-22

**Authors:** Kaili Wang, Mingtao Wang, Zixin Wan, Tao Shen

**Affiliations:** 1 Faculty of Information Engineering and Automation, Kunming University of Science and Technology, Kunming 650500, China; wangkaili0110@stu.kust.edu.cn (K.W.); wangmingtao@stu.kust.edu.cn (M.W.); wanzixin@stu.kust.edu.cn (Z.W.); 2 Yunnan Key Laboratory of Computer Techologies Application, Kunming 650500, China; 3 Yunnan Mechanical and Electrical Vocational and Technical College, Kunming 650500, China

**Keywords:** binary transformer, distribution alignment, knowledge distillation, distribution correction

## Abstract

Transformer is a powerful model widely used in artificial intelligence applications. It contains complex structures and has extremely high computational requirements that are not suitable for embedded intelligent sensors with limited computational resources. The binary quantization technology takes up less memory space and has a faster calculation speed; however, it is seldom studied for the lightweight transformer. Compared with full-precision networks, the key bottleneck lies in the distribution shift problem caused by the existing binary quantization methods. To tackle this problem, the feature distribution alignment operation in binarization is investigated. The median shift and mean restore is designed to ensure consistency between the binary feature distribution and the full-precision transformer. Then, a knowledge distillation architecture for distribution correction is developed, which has a teacher–student structure comprising a full-precision and binary transformer, to further rectify the feature distribution of the binary student network to ensure the completeness and accuracy of the data. Experimental results on the CIFAR10, CIFAR100, ImageNet-1k, and TinyImageNet datasets show the effectiveness of the proposed binary optimization model, which outperforms the previous state-of-the-art binarization mechanisms while maintaining the same computational complexity.

## 1. Introduction

Smart sensors have garnered widespread attention from researchers due to their powerful data acquisition and processing capabilities. By embedding deep learning models, sensors have achieved significant results in the field of artificial intelligence, including image classification [1,2,3], object detection [4,5], and semantic segmentation [6,7]. At present, a popular deep learning model is transformer [8], which has a powerful global feature representation ability compared with the traditional local convolutional neural networks (CNNs). Therefore, the fine architecture of transformer based on an attention mechanism and multi-layer perceptron has been proposed to solve complex problems, such as large language models (LLMs). However, transformer also introduces deployment constraints due to its large model size and extremely high computational burden, especially for embedded sensors with limited resources and insufficient computational power [9].

Quantization technology [10] has been considered the most effective approach for reducing the amount of calculations in transformer. The existing methods can be categorized into two categories: (1) high-bit quantization (16 bit, 8 bit) [11,12] and (2) low-bit quantization (4 bit, 2 bit, 1 bit) [13,14,15]. Evidently, binarization is an extreme form of quantization with weights, and activation values are −1 or +1 of 1 bit, which can minimize the model size and computational cost to the greatest extent. Nonetheless, at present, most binary neural networks (BNNs) are based on CNN architectures, with few studies on complex transformer architectures.

**Figure 1:**
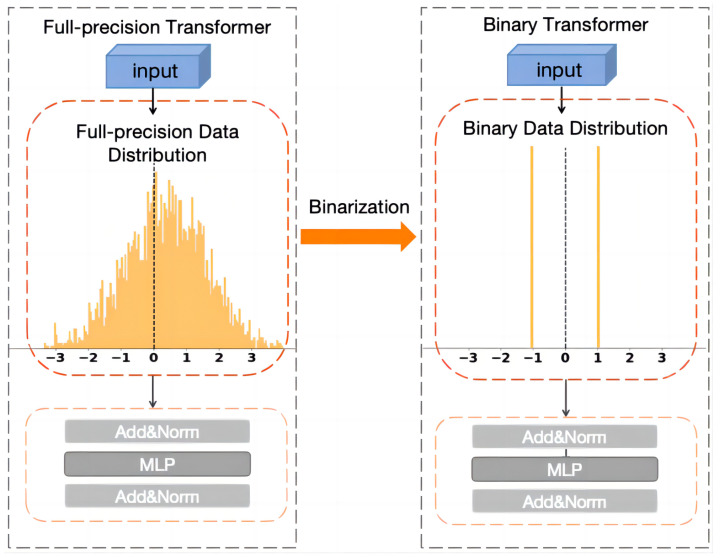
Distribution deviation in binarization.

In summary, it is still a challenging task to research the binary transformer so that its performance matches full-precision models to the maximum extent. The main reason for this is that simple binarization operations on attention values will lead to significant information loss, resulting in the inaccurate characterization of feature relationships [16]. Most existing methods attempt to maximize information entropy by adjusting network parameters [17], thereby enhancing their feature representation capabilities. Currently, many solutions have been studied under the constraint of maximum information entropy, such as weight reshaping [18] and minimization of gradient error [19]. Nevertheless, due to changes in the parameter structure, the feature distribution of binary data is significantly different from that of full-precision networks, as shown in Figure 1. The deviation of these feature distributions will cause larger feature representation errors with the stacking of attention blocks and eventually reduce the inference performance of the model.

Based on the above observations, maximum information entropy is the fundamental requirement for the binary transformer. On the other hand, how to maintain the feature distribution of the binary transformer as consistently as possible with the full-precision network is an issue that urgently needs to be solved in this field. Therefore, this paper investigated the alignment and correction of distribution for binary transformer (ACD-BiT). The contributions of this paper are summarized as follows.

1. A distribution alignment binarization method is proposed that addresses the problem of binary feature distribution deviation by median shift and mean restore. Distribution alignment binarization was performed on attention and full-connection layers, and it was further discussed that the effective binary operator is still under a constraint of maximum information entropy.
2. This paper introduces a knowledge distillation architecture for distribution correction. The teacher–student structure is built according to the full-precision and binary transformers. By performing the counterpoint distillation on the query Q and key K in attention and combining the label output of the full-precision teacher network to calculate the cross-entropy loss of the binary student network, the integrity and accuracy of binary transformer data are ensured.
3. Combining distribution alignment and distillation correction, we propose a novel transformer framework, ACD-BiT. While ensuring maximum information entropy, the feature distribution is consistent with the full-precision data to the greatest extent. To evaluate the effectiveness of the proposed model, experiments are conducted on various datasets, including CIFAR10, CIFAR100, ImageNet-1k, and TinyImageNet. The results show that the proposed model outperforms most of the existing mechanisms. This proves that both information entropy and data distribution are important factors affecting the performance of the binary transformer.

## 2. Related Works

In this section, advanced lightweight transformers are briefly reviewed. The current binary operators are also discussed to illustrate the key bottlenecks in binarization of deep networks.

### 2.1. Lightweight Transformers

Quantization can effectively reduce the model size and computational costs, replacing expensive floating-point operations with simple fixed-point operations. Therefore, the adoption of quantization techniques for research on lightweight transformers has received increasing attention from researchers. For instance, Yuan et al. [13] constructed an 8-bit transformer, reducing the quantization error of activation values by using a dual uniform quantization method. Li et al. [20] investigated a log-int-softmax mechanism for inference to construct a 4-bit quantization model. Wang et al. [16] proposed a binary transformer with a hierarchical-adaptive architecture, which replaces expensive matrix operations with addition and bit-wise operations by switching between two attention modes.

Different bit quantizations can lead to various lightweight transformer models, and the application scenarios and performance of these models are also diverse. Binary transformer is a superior extreme compression model, characterized by its smaller storage space and faster computation speed, and has a wider range of applications. However, compared to CNNs, complex transformer architectures experience more severe performance degradation as the degree of quantization increases.

### 2.2. Binary Networks

Binary neural networks (BNNs) quantize both weights and activations to the smallest 1 bit, thereby significantly reducing model size and computational consumption. There are many optimizations for binary operators in deep neural networks. Liu et al. [21] changed the data distribution of weights and activations by reshaping point-wise convolutions and balancing activations. Tan et al. [22] designed the gradient approximation function to reduce the gradient error in the back-propagation. Qin et al. [18] introduced a novel teacher–student network structure for knowledge distillation, thereby further optimizing binary neural networks. However, the aforementioned studies all aim to reduce the information loss of binary operators by optimizing the network structure.

In fact, differentiation in deep models can lead to extreme discrepancy of data distribution between full-precision and binary networks, making it difficult to maximize the performance of 1-bit networks. Although there have been some studies on data distribution bias issues, research on binary transformers is lacking [10,14].

## 3. Methods

This section first reviews the basic theory of binary neural networks. Then, the distribution alignment and distillation correction of two optimization modules are integrated to give the overall architecture of the novel binary transformer (ACD-BiT). Finally, a detailed study is conducted on the two key technologies: distribution alignment (Da) and distribution correction (Dc).

### 3.1. Preliminary

**Binary Neural Networks (BNNs).** As the most extreme case of quantized networks, weights and activations are constrained to be +1 and −1 after binary operations. For full-precision inputs, the sign function is used to perform binary processing to generate the corresponding binary output results [23]. The forward-propagation process of the sign function in the binarized network can be expressed as the following formula:

(1) $$x_{b}=Sign\left(x\right)=\left\{\begin{array}{c} +1,\;if\;x\geq 0\\ -1,\;otherwise \end{array}\right.$$

During the back-propagation process, usually, a straight-through estimator (STE) is used to solve the non-differentiability problem of the sign function [24]. Specifically, an approximation function is used instead of the sign function to back-propagate the gradient to the full-precision weights or activations for updating. Back-propagation can be represented as

(2) $$\frac{\partial L}{\partial x}=\left\{\begin{array}{cc} \frac{\partial L}{\partial x_{b}} & ,\;if\;|x|\leq 1\\ 0 & ,\;otherwise \end{array}\right.$$

where L is the cost function for the minibatch. To minimize the quantization errors, BNNs introduce a full-precision scaling factor α, as follows:

(3) $$\alpha =\frac{{∥x∥}_{\ell_{1}}}{n},\;x\approx \alpha x$$

where ${∥\cdot ∥}_{\ell_{1}}$ denotes the $\ell_{1}\text{-}$norm, and n is the dimension of x.

Then, the sign function is applied in forward propagation, and STE is used to obtain the derivatives in back-propagation. For deep neural networks, let W and A represent the full-precision weight and activation, respectively. $W_{b}$ and $A_{b}$ represent the quantized binary weight and activation. Thus, the conventional convolution operation can be represented as

(4) $$A⊗W=A_{b}⊕W_{b}\cdot \alpha_{a}\alpha_{w}=\left(Sign\left(A\right)⊕Sign\left(W-Mean\left(W\right)\right)\right)\cdot \alpha_{a}\alpha_{w}$$

where ⊗ denotes the convolution operation, ⊕ denotes bit-wise XNOR and bit-count calculations, Mean(·) is the mean value, and $\alpha_{a}$ and $\alpha_{w}$ are the scaling factors for activation and weight.

**Binary Transformer.** The transformer network mainly consists of N encoder blocks, each containing multi-head attention (MHA) and feed-forward network (FFN) modules. The input data $H=R^{n\times d}$ are first passed through the binarized linear layer to obtain the batch-normalization of queries Q, keys K, and values V, which serve as the input data for the MHA module. Then, the attention score A is calculated as follows:

(5) $$A_{score}=\frac{Sign\left({\mathrm{BN}}_{Q}\left(H\right)\right)⊕Sign{\left({\mathrm{BN}}_{K}\left(H\right)\right)}^{T}}{\sqrt{d_{h}}}$$

where ${\mathrm{BN}}_{Q}$ and ${\mathrm{BN}}_{K}$ denote different binarized linear layers for Q and K, respectively. $d_{h}=\frac{d}{N_{h}}$, where d is the dimension of features and $N_{h}$ denotes the attention heads.

Then, the binary attention feature matrix performs feature enhancement on the binarized value matrix V to obtain the feature mapping output matrix P of MHA, as follows:

(6) $$P=RPReLU({\mathrm{BN}}_{attention}\left(Softmax\left(A_{score}\right)\cdot Sign\left({\mathrm{BN}}_{V}\left(H\right)\right)\right))$$

where ${\mathrm{BN}}_{V}$ denotes the binarized linear layer of V, and RPReLU(·) is the activation function.

### 3.2. Overall Framework of Transformer Binarization

**Figure 2:**
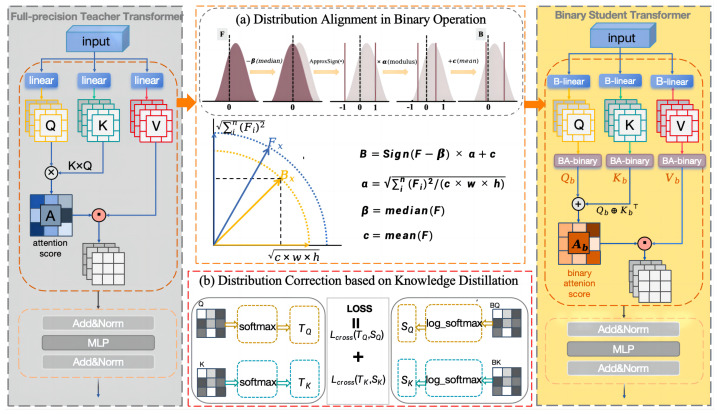
Overall framework of binary transformer.

In this paper, we propose a novel binary strategy based on distributed alignment for transformer; the overall architecture is shown in Figure 2. The core of the binary strategy is to study the attention mechanism in transformer and perform binary operations on the feature values Q, K, and V, thereby effectively reducing the model size and computational burden. First, to overcome the problem of feature distribution deviation caused by classical feature reshaping binary strategies, a novel distribution alignment in binary operation is proposed, as shown in Figure 2a. Then, in order to further correct the binarized feature distribution, a teacher–student structure based on a full-precision teacher model and a binary student model is constructed, as shown in Figure 2b. By effectively combining the above two methods, while maximizing information entropy, the binary feature distribution is maximally consistent with the original full-precision features, enhancing the feature representation capability of the binary attention.

### 3.3. Distribution Alignment in Binary Operation

The two core requirements of binary neural networks are to minimize information loss and quantization errors. In this section, the binary strategy of distribution alignment based on median shift and mean restore is comprehensively introduced, and the alignment method for weights and activations that simultaneously satisfy information loss and quantization error minimization is presented. Then, the specific algorithm process in the attention mechanism is given.

#### 3.3.1. Binary Strategy for Distributed Alignment

Binary neural networks use 1 bit to represent raw floating-point numbers, and the quantization can be formulated as

(7) $$Q\left(x\right)=\alpha B_{x}$$

where x denotes full-precision data, α represents scalars of binary values, and $B_{x}$ are binary values, as shown in formula (1). Then, in forward propagation, the vector multiplication of weights and activations can be represented as

(8) $$F_{a}\cdot F_{w}=Q\left(a\right)\cdot Q\left(w\right)=\left(B_{a}⊕B_{w}\right)\cdot \alpha_{a}\alpha_{w}$$

where F and B denote full-precision and binary, and ⊕ denotes bit-wise XNOR and bit-count calculations.

**Minimizing information loss.** Due to the binarization process, which compresses the weights and activations extremely to 1 bit, parameter representation capabilities are limited. In order to retain as much of the original full-precision information as possible, the mutual information [25] between the binary representation and the full-precision representation should be maximized; that is,

(9) maxI(F;B)=H(B)−H(F/B)

where B denotes the information entropy, and H(F/B) denotes the conditional entropy of B given F. Since the binary function is deterministic, H(F/B)=0. That is, maximizing mutual information is equivalent to maximizing information entropy, and the formula is as follows:

(10) $$\max I\left(F;B\right)=\max H\left(B\right)=-\sum_{b}P\left(b\right)\log P\left(b\right)$$

where P(b) denotes the probability of taking the value b. Combined with the sign function Formula (1), $b\in \left\{-1,1\right\}$. Then, the information entropy can be expressed as follows:

(11) $$\begin{array}{cc} H(B) & =-P(1)\log P(1)-P(-1)\log P(-1)\\ \; & =-P(1)\log P(1)-(1-P(1))\log(1-P(1)) \end{array}$$

Then, by taking the derivative of H(B) with respect to P(1):

(12) $$\frac{dH(B)}{dP(1)}=-\left(\log P\left(1\right)+\frac{1}{\ln2}\right)+\left(\log\left(1-P\left(1\right)\right)+\frac{1}{\ln2}\right)=\log\left(\frac{1-P(1)}{P(1)}\right)$$

Let $\frac{dH(B)}{dP(1)}=0$; the maximum of H(B) is obtained, and then P=0.5. This indicates that the binarized data should present a uniform distribution, and their probability density function is shown as follows:

(13) $$P\left(b\right)=\left\{\begin{array}{c} 0.5,\;if\;b=+1\\ 0.5,\;if\;b=-1 \end{array}\right.$$

**Figure 3:**
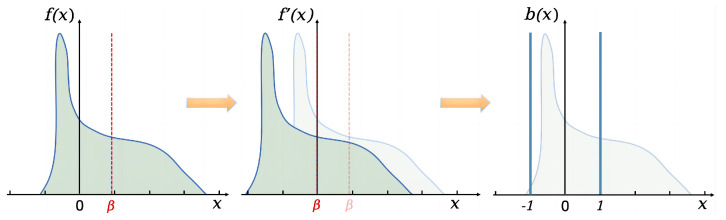
Median shift binarization process. f(x) and b(x) represent the full-precision and binary distributions; the middle $f^{\prime }\left(x\right)$ represents the shifted full-precision distribution; and β is the median.

Therefore, the binarization of attention weights and activations needs to satisfy the maximization of information entropy, achieve a uniform distribution of binary values, and thereby enhance the representational ability of attention. The uniform distribution quantization process is shown in Figure 3.

Before binarization, to ensure that the number of features on both sides of the zero mean is the same, the median value is subtracted from the full-precision, i.e., $\tilde{F}=F-Median\left(F\right)$. Thus, the data are centered around zero with an equal number of features on each side. Then, through the binarization sign function, the following formula can be obtained:

(14) $$Sign\left(f^{\prime }\left(x\right)\right)=\left\{\begin{array}{c} -1,\;if\;x<0\\ +1,\;if\;x\geq 0 \end{array}\right.$$

The uniform binary distribution is achieved by the binarization operation after the median shift, resulting in minimal information loss after quantization. However, the binary distribution after translation shows a significant difference from the original full-precision distribution, leading to severe quantization errors.

**Minimizing quantization error.** The minimization of information loss carried by binary neurons improves the representation ability of attention features; however, in order to ensure superior binary neural networks, it is necessary to further minimize the quantization error [26], and the formula is defined as follows:

(15) $$\min Q_{e}\left(X\right)={∥F(X)-Q(X)∥}^{2}$$

where F(X) represents the full-precision feature distribution, Q(X) represents the binary feature distribution, and quantization error $Q_{e}\left(X\right)$ is used to measure the feature distribution difference between full-precision and binary.

**Figure 4:**
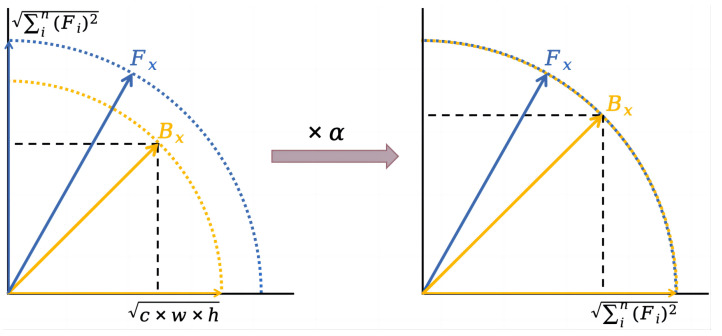
Schematic diagram of the modulus length alignment. $F_{x}$ and $B_{x}$ represent the full-precision and binary vectors, and α is the scaling factor.

To ensure that the distribution of the binary feature $B_{x}$ remains consistent with that of the full-precision feature $F_{x}$, first, it is necessary to ensure that the modulus length is the same before and after quantization. For the full-precision vector $F_{x}\in R^{c\times w\times h}$, its modulus length is defined as $\sqrt{\sum_{i}^{n}{\left(F_{i}\right)}^{2}}$, while for the binary vector $B_{x}\in {\left\{-1,1\right\}}^{c\times w\times h}$, its modulus length is fixed equal to $\sqrt{c\cdot w\cdot h}$. Therefore, a scaling factor $\alpha =\sqrt{\sum_{i}^{n}{\left(F_{i}\right)}^{2}/c\cdot w\cdot h}$ needs to be introduced to make the quantized modulus length consistent with the full-precision one by scaling the binary $B_{x}$, thereby reducing the quantization error. The schematic diagram of the magnitude scaling is shown in Figure 4.

Subsequently, the translation operation carried out to maximize information entropy leads to the center position of the binary data distribution ($C_{pos}=0$) being inconsistent with the full-precision one ($C_{pos}=Mean\left(F_{x}\right)$). Therefore, it is necessary to introduce a bias factor $c=Mean(F_{x})$ to restore the binarization $B_{x}$. Finally, the binary feature $B_{x}^{\prime }$ that minimizes the quantization error is obtained:

(16) $$B_{x}^{\prime }=\alpha \cdot B_{x}+c=\sqrt{\frac{\sum_{i}^{n}{\left(F_{i}\right)}^{2}}{c\cdot w\cdot h}}\cdot B_{x}+Mean\left(F_{x}\right)$$

In summary, by performing median shifting, binary quantization of the sign function, modulus scaling, and mean restoration, the two core requirements of the binary network are achieved: minimizing information loss and minimizing quantization error. After applying the distribution alignment binary strategy, the binary features obtained are optimized for maximize information entropy while the feature distribution matches the full-precision features to the maximum extent, thereby significantly enhancing the feature representation capability of binary attention, as shown in Figure 2a.

#### 3.3.2. Algorithm Flow in Attention

The transformer block consists of two major components: multi-head self-attention (MHSA) and multi-layer perceptron (MLP). The input data come from the hidden layer states $X=R^{n\times d}$, which are d tokens of length n. The query (Q), key (K), and value (V) that the attention calculation depends on are obtained through different binarized linear layers. The computation of the input features can be represented as

(17) $$Q=D_{a}{\text{-linear}}_{q}\left(X\right),\;K=D_{a}{\text{-linear}}_{k}\left(X\right),\;V=D_{a}{\text{-linear}}_{v}\left(X\right)$$

where $D_{a}{\text{-linear}}_{q}$, $D_{a}{\text{-linear}}_{k}$, and $D_{a}{\text{-linear}}_{v}$ denote three different binary linear layers based on distribution alignment for Q, K, and V, respectively. Then, distribution alignment binarization is performed on the linear output features to obtain input features for the calculation of attention feature relations. The attention score A is calculated as follows:

(18) $$A_{score}=\frac{B_{da}\left(Q\right)⊕B_{da}{\left(K\right)}^{T}}{\sqrt{d_{h}}}$$

where $B_{da}$ represents the distribution alignment binarization operation, and $d_{h}$ denotes the feature dimension of the multi-head attention.

Then, the softmax function is used for normalization to obtain the feature probability matrix $A=Softmax(A_{score})$. The feature enhancement operation is performed by multiplying A with the binarized feature matrix $B_{v}=B_{da}\left(V\right)$, obtaining the feature mapping output. To better preserve the original feature information, the Q, K, and V of linear layer outputs are further added. The final attention output is as follows:

(19) $$P_{b}=RPReLU\left({\mathrm{BN}}_{attention}\left(A\cdot B_{v}\right)+Q+K+V\right)$$

For a specific transformer block, the binary quantization process is summarized in Algorithm 1.
**Algorithm 1** Binary quantization in Attention1:**Input:** Hide states feature matrix $X\;\in \;R^{n\times d}$ (d tokens of length n), weight matrix $W\;\in \;R^{d_{in}\times d_{out}}$.2:**Output:** Attention feature mapping P.3:Binary linear layer:        $B_{x}=\alpha_{x}\cdot ApproSign\left(X-\beta_{x}\right)+c_{x}$        $B_{w}=ApproSign\left(\left(W-\beta_{w}\right)/\alpha_{w}\right)$        $B\left(X\right)=Linear\left(B_{x},B_{w}\right)$        $D_{a}\text{-linear}=RPReLU\left({\mathrm{BN}}_{attention}\left(B\left(X\right)+R\left(X\right)\right)\right)$        get the feature matrices:                $Q=D_{a}{\text{-linear}}_{q}\left(X\right),\;K=D_{a}{\text{-linear}}_{k}\left(X\right),\;V=D_{a}{\text{-linear}}_{v}\left(X\right)$4:Compute binary features:       $Q_{b}=\alpha \cdot ApproSign\left(Q-\beta \right)+c$       $K_{b}=\alpha \cdot ApproSign\left(K-\beta \right)+c$       $V_{b}=\alpha \cdot ApproSign\left(V-\beta \right)+c$5:Feature relation $R_{b}=\left(Q_{b}⊕K_{b}^{T}\right)/\sqrt{d_{h}}$6:Attention feature $A_{s}=Softmax\left(R_{b}\right)\cdot V_{b}$7:**return** Feature mapping $P=RPReLU({\mathrm{BN}}_{attention}\left(A_{s}\right)+Q+K+V)$.

### 3.4. Distribution Correction Based on Knowledge Distillation

Knowledge distillation is an essential supervised method for training quantized networks [27]. The teacher–student network structure, constructed with full-precision and binary models, is well fit regarding the performance difference between binary and full-precision models [28]. Therefore, in order to maintain the distribution consistency of the attention binary features as much as possible, in this paper, we further introduce a distribution correction method based on knowledge distillation, which performs counterpoint distillation on the query Q and key K and calculates the cross-entropy loss of the binary student network based on the label output of the full-precision teacher network, thereby further correcting the feature distribution of the binarization.

The attention feature matrices Q and K were extracted in a layerwise manner from the full-precision teacher network, and the mean squared error (MSE) was used as the loss function to measure the difference between the binary student network and the teacher network in the corresponding features [29]. The expression for feature counterpoint distillation is as follows:

(20) $$L_{Q}=\sum_{l=1}^{l}MSE\left(Q_{l},Q_{Tl}\right),\;L_{K}=\sum_{l=1}^{l}MSE\left(K_{l},K_{Tl}\right)$$

Let $P_{t}$ be the output prediction of the teacher network, which is used for student network learning to generate soft labels, and let $P_{s}$ be the output prediction of the student network. Then, based on the cross-entropy loss function, the following distillation formula can be obtained:

(21) $$L_{\text{hd-dist}}=\alpha L_{CE}\left(\Psi \left(P_{s}\right),y\right)+\beta L_{CE}\left(\Psi \left(P_{s}\right),y_{t}\right)$$

where y denotes the true label, $y_{t}=arg\max\left(P_{t}\right)$ indicates the label derived from the teacher network, Ψ(·) represents the softmax function, $L_{CE}$ is the cross-entropy loss function, and α and β denote the weight scaling factors.

Therefore, the distillation architecture proposed in this paper first corrects the feature matrix of attention by distillation and then minimizes the loss of the student network with respect to the true labels, while also minimizing the label loss of the teacher network. The distillation objective function can be expressed as follows:

(22) $$L_{golbal}=\min\left(L_{Q}+L_{K}\right)+\min\left(S,R\right)+\min\left(S,T\right)$$

Since the performance of the full-precision teacher network is superior to that of the binary network, the proposed distillation network of the teacher–student architecture selects α=0.9 and β=0.1. By increasing the full-precision teacher correction weights to further enhance the representational ability of the binary neural network, the performance of the student network can be made to approach full-precision to the greatest extent.

## 4. Experiments

To assess the performance of the proposed ACD-BiT, a series of experiments were conducted on four widely used benchmark datasets: CIFAR10, CIFAR100, ImageNet-1k, and TinyImageNet. First, the experimental datasets, evaluation metrics, and experiment setting were introduced. Second, the loss gradient update parameter of the distillation architecture was tuned and verified. Then, compared with some representative binary transformers, the performance of the model was verified. Then, ablation experiments were carried out on distribution alignment and distillation correction, respectively, to evaluate the effectiveness of the proposed model. Lastly, a series of visualization experiments were carried out to further verify the representation ability of the model. BinaryViT [30], the classical binary network of PVT architecture, was selected as the baseline model, and all parameters were consistent with the original model except the transformer block.

### 4.1. Implementation Details

In the experiments, ACD-BiT was based on a binary transformer implemented in the PyTorch framework using novel distributed reduction and distillation correction methods. The experiments were conducted on a server equipped with two Tesla A100 GPUs, and the operating system was running on Ubuntu 20.04.

**Benchmark Datasets.** To evaluate the effectiveness of the proposed model, the experiments were verified on four datasets.

- CIFAR10 [31] consists of 60,000 color images of size 32 × 32 divided into 10 categories with 6000 images each. It contains 50,000 training images and 10,000 test images, divided into five training batches and one test batch (each batch contains 10,000 images).
- CIFAR100 [31] contains 100 categories with 600 images per category. Each category is divided into 500 training images and 100 test images. The 100 categories are organized into 20 superclasses such that each image has a “fine” label and a “coarse” label.
- ImageNet-1k [32] consists of 1,281,167 training images and 50,000 validation images covering 1000 categories. Each image is carefully labeled and contains diverse and refined categories, making this one of the most complex large-scale image classification datasets.
- Tiny-ImageNet [32] is extracted from the ImageNet dataset and contains 100,000 images of 200 categories (500 for each class) downsized to 64 × 64 colored images. There are 500 training images and 50 test images for each class.

**Evaluation Metrics.** The main evaluation metrics of the experiment are the image classification accuracy of Top-1 and Top-5, which characterize the accuracy of the first category and the first five categories predicted by the model classification, respectively.

**Experiment Setting.** The weights and activations, including Q, K, V and projection layers of the attention module, were binarized by distribution alignment to realize binary attention. In all experiments, the images were uniformly scaled to 224 × 224, the batch size was set to 128, and the model was trained using the AdamW optimizer with an initial learning rate of $10^{-4}$ and a weight decay of 0.05. For fair comparison, all experiments were trained for 300 epochs. In the training process, the ViTForImageClassification model was first used to train the teacher model with full precision and then load the optimal weight of the teacher model into the feature values in intermediate layers and prediction outputs of the student network for distillation correction.

### 4.2. Hyperparameter Tuning Validation

In the proposed ACD-BiT, the distillation correction method adopted sets the loss gradient update scaling factor for the full-precision teacher network and the binary student network, which significantly affects the overall performance of the model. Specific hyperparameter tuning and validation experiments were conducted on CIFAR10 and CIFAR100, as shown in Table 1.

The results obtained from the experiments show that as the loss calculation ratio of the full-precision teacher network continues to increase, the overall performance of the model continuously improves. Moreover, the experimental results on CIFAR10 and CIAFAR100 datasets are consistent. It is evident that by increasing the full-precision teacher correction weights, the representational ability of the binary neural network is further enhanced, thereby making the performance of the student network as close to full precision as possible. Therefore, a distillation network with a teacher–student architecture is proposed with α=0.9 and β=0.1.

### 4.3. Comparative Experiments and Analysis

To quantitatively evaluate the performance of the proposed binary transformer based on distribution alignment and correction, a series of quantitative methods were compared and analyzed against classic models on CIFAR-10, CIFAR-100, and ImageNet-1k. This includes the overall performance and computational cost of the models.

#### 4.3.1. Performance Comparison

As shown in Table 2, the performance degradation of the model increased with the continuous increase in the quantization gradient. The proposed method, ACD-BiT, effectively improved the performance of the model while weights and activations both reached the ultimate compression rate of 1 bit. Compared to the baseline model BinaryViT, Top-1 and Top-5 accuracy were improved by 5.37% and 0.63% on CIFAR10 and 1.65% and 2.5% on CIFAR100, respectively. Compared with the state-of-the-art model BinaryFormer, the classification accuracy also improved by 2.65% and 0.37% on CIFAR10 and 1.22% and 2.7% on CIFAR100, respectively. For the large-scale dataset ImageNet-1k, the proposed method had higher inference accuracy. It was 5.43% and 3.39% higher than BinaryViT on Top-1 and Top-5 and 4.30% and 0.79% higher than BinaryFormer, respectively.

#### 4.3.2. Efficiency Comparison

To further illustrate the computational cost of the proposed method, Table 2 also show the model size and computational cost comparison of different methods. The calculation method for model size is the number of model parameters multiplied by four, which is used to evaluate the cost of storage space required for model deployment. The total number of operations (OPs) is calculated using binary operations (BOPs) and floating-point operations (FLOPs), that is, OPs = BOPs/64 + FLOPs, which is used to evaluate the computational cost of the method. It is evident that the memory occupation of ACD-BiT was only 5.8% of the full-precision network, and the computational overhead was reduced by 47.8%. Compared with all the binarization methods, ACD-BiT had the same memory and computational complexity but achieved further improvement in accuracy.

#### 4.3.3. Robustness Comparison

**Figure 5:**
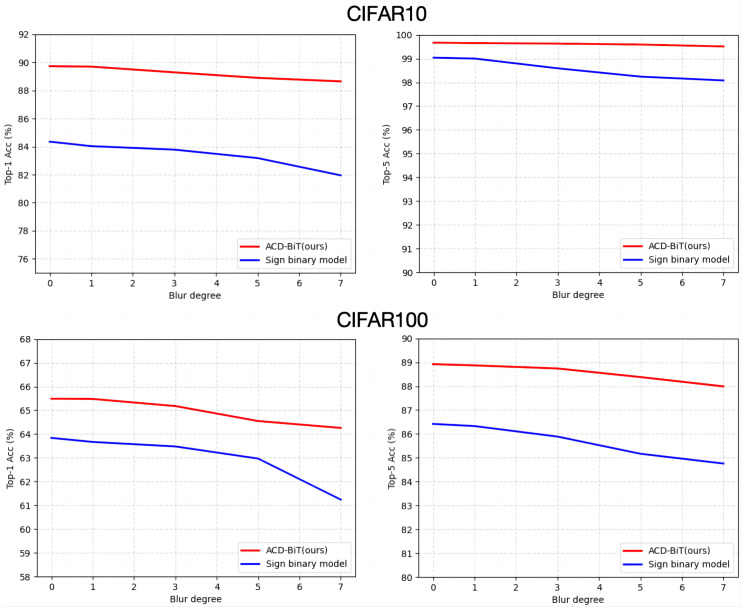
Performance on different blur degrees.

In order to further evaluate the robustness of the model, Gaussian blur was used to perform varying degrees of blur operations on the CIFAR10 and CIFAR100 training datasets. The experiment compared the proposed distribution alignment and correction binary transformer with the classic Sign binary model, with the experimental results shown in Figure 5.

As can be clearly seen from the curve graph, the model performance decreased to a certain extent as the blur gradient increased. The ACD-BiT architecture (indicated by the red curve) proposed in this paper decreased from 89.72% to 88.31% in Top-1 accuracy, and the performance was only reduced by 1.41%. In contrast, the performance of the traditional Sign model decreased by 5.8%, which is a significant drop. It is evident that the overall performance of ACD-BiT was superior to that of the Sign model, and the decline in its performance tends to be gentle with the increase in the blur degree, reflecting good robustness.

#### 4.3.4. Comparative Experimental Analysis

Comparative experimental results indicate that the binary quantization ACD-BiT model achieved the best performance on the classic CIFAR image classification datasets and also reached the optimal accuracy on the complex larger-scale dataset ImageNet-1k. Extreme binary quantization significantly reduces accuracy due to the loss of information. This paper combines distribution alignment and distillation correction methods to maximize the restoration of binary feature distributions, improving inference efficiency and further enhancing the accuracy of model inference. Compared to other binarization methods, ACD-BiT adopts an efficient binarization method based on median shift and mean restoration, directly operating on raw full-precision data to avoid the restoration process during inference. This method maximizes information entropy while maintaining a consistent feature data distribution with the original data, thereby improving the accuracy of model inference. In addition, the distillation correction method uses the full-precision teacher network to distill the middle layers features and prediction output of the student network so as to further ensure the consistency of data distribution and enhance the capabilities of the binary network.

### 4.4. Ablation Experiments and Analysis

This section describes the ablation experiments that were conducted on the two core components of the proposed model ACD-BiT: distribution alignment and distillation correction. It fully verifies the effectiveness of the two modules on model performance and then offers a specific analysis according to the experimental results.

#### 4.4.1. Effectiveness of Two Modules in Binary Strategy

The ablation experiment results are shown in Table 3. The binarization method of ACD-BiT achieved extreme compression to 1 bit in terms of weights and activations, with distribution alignment (Da) and distillation correction (Dc) as the two main modules. First, applying the binary strategy of distribution alignment (Da) alone, the model Top-1 accuracy was improved by 3.26%, 0.78%, and 1.99% on the CIFAR10, CIFAR100, and TinyImageNet datasets, respectively. Meanwhile, the Top-5 accuracy was increased by 0.23%, 1.39%, and 0.78%, respectively. Then, only using distillation-based distribution correction (Dc), the Top-1 accuracy of the model also increased by 1.86%, 0.46%, and 0.69% on the three datasets, and the Top-5 accuracy was improved by 0.02%, 1.33%, and 0.15%, respectively. It is evident that the combination of the above two methods optimized model performance to the greatest extent, achieving the highest precision in binarization.

#### 4.4.2. Ablation Experimental Analysis

Through the ablation experiments, the effectiveness and necessity of the distribution alignment (Da) and the distillation correction (Dc) methods in the proposed binary transformer were effectively verified. The binary strategy proposed in this paper effectively solved the problem of feature data distribution deviation caused by information entropy maximization in binary neural networks. The distribution alignment method directly operated on the original full-precision network while maintaining the diversity of information flow data, thereby improving the inference efficiency and reducing information loss caused by quantization. Through median shift and mean restore operation, the original full-precision data distribution was maximally recovered while ensuring the maximum information entropy of the binary data, thereby enhancing the representational capability of the model. The correction method based on knowledge distillation constructed a teacher–student structure by training a full-precision teacher network and a binary student network. The counterpoint distillation of the middle layer features of the student network was combined with the prediction output cross-entropy loss calculation to further restore the binary data distribution and improve the reasoning ability of the model. In summary, binarized models combined with distribution alignment and correction can effectively improve inference performance.

### 4.5. Visualization Analysis Experiments

In this section, to further verify the effectiveness of ACD-BiT, the binary feature distribution of feature values, the feature relationship mapping distribution, and the self-attention feature mapping are visualized on the TinyImageNet dataset, respectively.

#### 4.5.1. Effectiveness of Distribution Alignment

**Figure 6:**
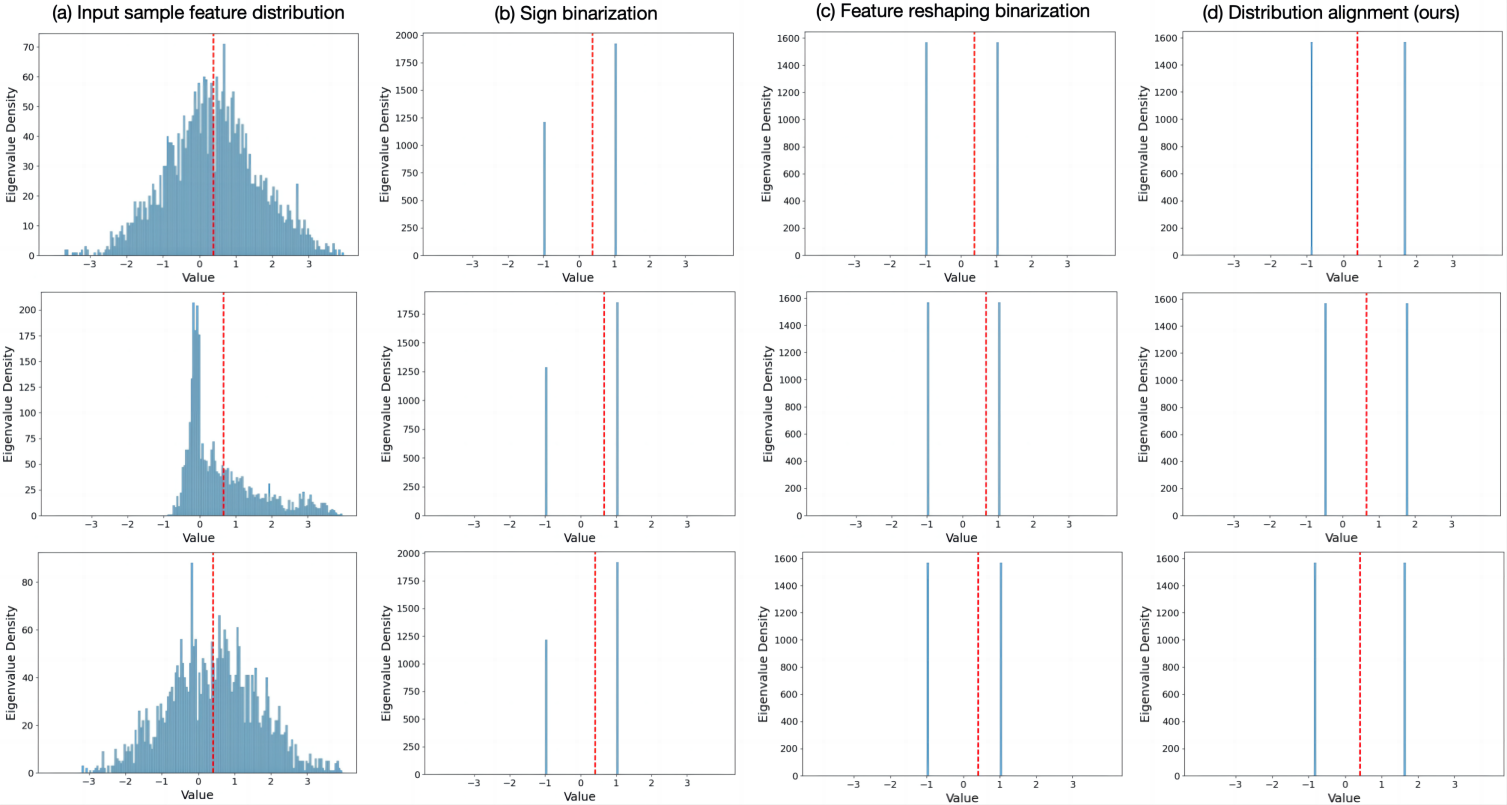
Feature distributions of utilizing diverse binary strategies. (**a**) is the full-precision feature distribution of the input sample, with the red dashed line indicating the mean symmetry axis of the feature distribution. (**b**) represents the traditional binary strategy based on Sign function. (**c**) is the binary strategy of feature reshaping adopted to maximize information entropy. (**d**) is the distribution alignment binary strategy proposed in this paper to achieve feature distribution restoration. The symmetry axis of the input sample is used as the criterion to evaluate the performance of distribution alignment.

The binary strategy based on median shift and mean restore can maximize the consistency between the binary feature distribution and the original full-precision feature distribution. Figure 6 illustrates the comparison of feature distributions under different binary strategies, using the symmetric axis of the full-precision feature distribution (red dashed line) as the evaluation criterion. It is evident that the Sign binary leads to severe information loss, and there is a large quantization error compared to the full-precision data. The feature reshaping binary aims to make −1 and 1 as balanced as possible, maximizing information entropy, but the feature distribution deviates from the symmetric center and has a large deviation from the original full-precision feature. Our distribution alignment and correction binary effectively restores the original full-precision feature distribution while satisfying the maximum information entropy. Its binary feature sets are aligned based on the symmetric axis, thereby comprehensively enhancing the reasoning ability of the binary network.

#### 4.5.2. Performance of Feature Representation

**Figure 7:**
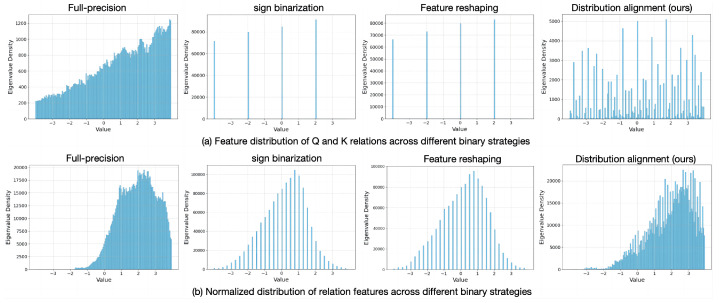
Distribution comparison of feature relation mapping under different binary strategies. The top part is the feature distribution of the relationship between feature query and feature key, and the bottom part is the normalized results of the feature relationships.

To further illustrate that the distribution alignment binary strategy can maximize the inference capability of the model through the median shift and mean restore methods, the feature distribution visualization of the attention feature relationship mapping and its normalized features was performed. The results are shown in Figure 7, and the relationship features of both Sign binarization and feature reshaping binarization show significant deviations from the full-precision feature distribution, indicating a severe decline in the representational capability of the feature relationships. Although feature reshaping maximized information entropy and increased the information capacity of models, the singleness of the information flow data also led to insufficient representation of feature relationships based on binarized features. It is obvious that the distribution alignment method greatly enhanced the representational capability of the feature relationships and that the normalized relationship mapping feature distribution remained consistent with the full-precision. From this, it can be seen that the distribution alignment binary strategy not only maximizes information entropy but also restores the full-precision data distribution while maintaining the diversity of the information flow data, thereby effectively enhancing the inference capability of the models.

#### 4.5.3. Attention Feature-Guided Visualization

**Figure 8:**
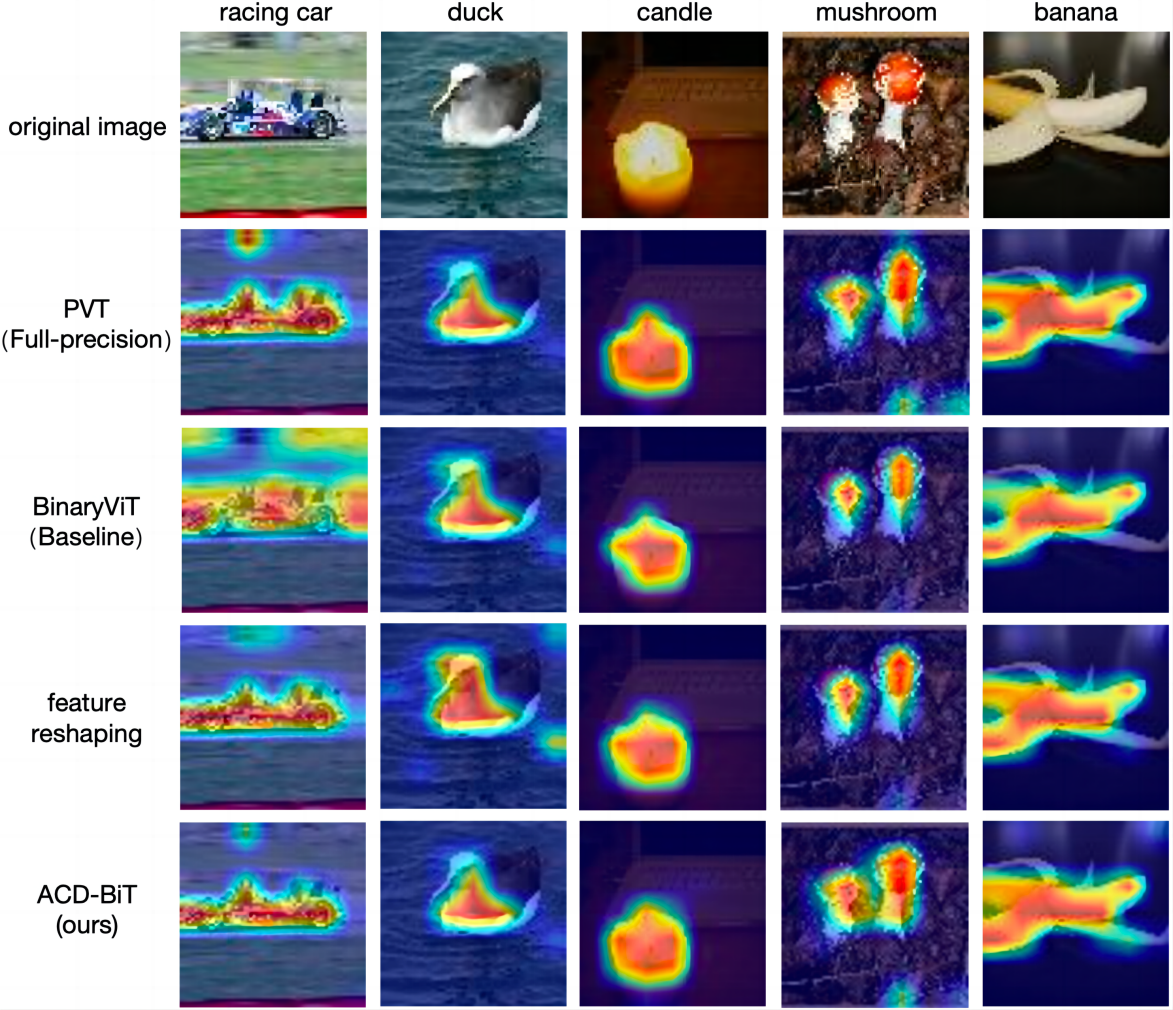
Heat map of self-attention feature mapping.

To analyze the inference ability of the proposed binary quantization model, feature mapping of the self-attention layer was used to generate the feature activation map [37]. As shown in Figure 8, it highlights the key areas of images that are most relevant to the specific classification prediction, demonstrating the image locations guided by the attention features of model. It is evident that the proposed ACD-BiT could accurately guide the network to focus on the critical areas of the predicted classification objects. Meanwhile, compared to the baseline BinaryViT and the feature reshaping binary quantization model, ACD-BiT paid more attention to areas containing target objects. The visualization results are consistent with the theoretical analysis, which demonstrated that the proposed binary quantization transformer has superior feature representation capability.

#### 4.5.4. Quantization Error Effect on Attention Matrix

**Figure 9:**
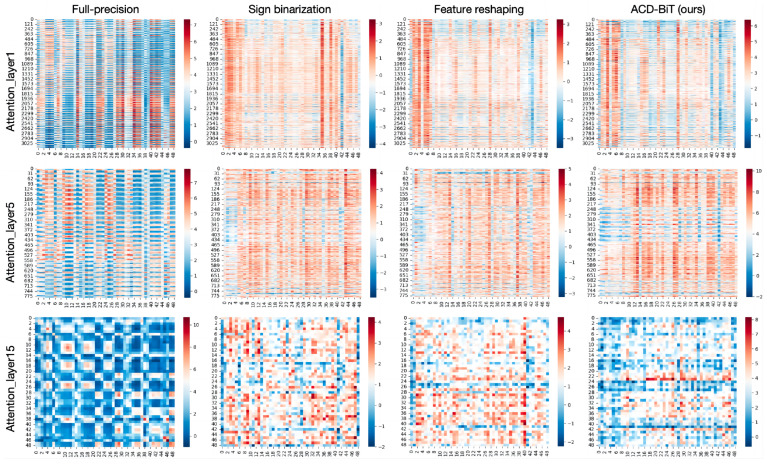
Visualization of attention feature maps across different stages of the model.

In order to minimize the computational consumption and deployment pressure, this paper proposes a binary quantization transformer. However, the binary quantization of feature information inevitably brings about representation errors. In order to analyze and compare the performance impact of quantization error on various layers of the model in more detail, the attention feature layer mapping was visualized, as shown in Figure 9. Compared with the full-precision feature map without quantization errors, the distribution of the quantized feature should be as consistent with the full-precision data as possible in diversity and accuracy. It is obvious that the binarized feature distribution of the traditional Sign function was relatively single. As the number of layers increased, the proposed ACD-BiT feature distribution was the closest to the original full-precision and had the strongest representation power.

In the future, in order to further improve the model performance of the binarized network, it is necessary to further study the feature properties and distribution characteristics of the model so as to make the binary network accuracy as consistent with the full-precision performance as possible.

#### 4.5.5. Visual Experimental Analysis

From the four types of visualization experiments, ACD-BiT combined with the distribution alignment based on median shift and mean restore and the distillation correction method based on the teacher–student structure achieved superior performance in terms of binary feature distribution, feature relationship mapping distribution, attention feature representation, and quantization error reduction.

The distribution alignment binary strategy directly performed median shift and mean restore on the full-precision data, ensuring that the quantized binary features maximize the information entropy while maintaining the feature distribution consistent with the full-precision data. Meanwhile, the diversity of information flow data was preserved through modulus scaling and restore, enhancing the feature representation capability. Subsequently, the full-precision teacher network further corrected the feature distribution of the binary student network, minimizing the quantization loss and further ensuring the completeness and accuracy of the data. Through the combination of Da and Dc, the inference ability of the binary transformer model was effectively improved.

## 5. Conclusions

Binary quantization transformers, which are more suitable for smart sensors, are seldom investigated. The key bottleneck is the distribution changes compared with full-precision networks, which is caused by existing binarization optimization methods. Motivated by this, the authors of this paper demonstrate improvements from two aspects. (1) The binarization was investigated through a distribution alignment operation. The median shift and mean restore methods were carefully designed to maintain the consistency of the data distribution. Then, the binarization of attention and full-connection were optimized through distribution alignment. It was also discussed whether the effective binary operator is still under the constraint of maximum information entropy. (2) A knowledge distillation architecture for distribution rectification was proposed. The teacher–student structure was chosen according to the full-precision and binary transformers. The feature distribution was further corrected by the counterpoint distillation of the attention middle layer features and the cross-entropy loss calculation of the prediction output. This guaranteed that the data distribution of binary transformers was as consistent with the full-precision models as possible. As a result, the experiments showed that our model was superior to most existing mechanisms. This means that information entropy and data distribution are all-important factors in binary transformers. Based on this study, a complex transformer model can be deployed in a variety of smart sensors with poor computation resources.

## Figures and Tables

**Table 1:** Validation experiment of loss gradient update scale factor for distillation correction method. α and β are the loss calculation ratios of the full-precision teacher network and the binary student network, respectively.

α/β	CIFAR10 (%)		CIFAR100 (%)	
	**Top-1**	**Top-5**	**Top-1**	**Top-5**
0.4/0.6	88.84	99.48	63.56	86.78
0.6/0.4	89.20	99.55	63.68	86.87
0.8/0.2	89.46	99.65	63.71	86.96
0.9/0.1	**89.72**	**99.67**	**65.49**	**88.92**

bolded numbers signify optimal results.

**Table 2:** Overall performance and computational consumption comparison of various quantization methods on the classic CIFAR and ImageNet-1k datasets. W and A denote the weight bit width and the activation bit width, respectively.

Methods	W/A	Model Size	OPs	CIFAR10 (%)		CIFAR100 (%)		ImageNet-1k (%)	
				**Top-1**	**Top-5**	**Top-1**	**Top-5**	**Top-1**	**Top-5**
ViT (Teacher) [1]	32/32	86.9 M	6.27 G	91.63	99.74	79.59	92.40	80.0	94.94
Mixed-Precision [11]	16/16	47.8 M	6.27 G	94.58	99.79	83.06	97.13	76.02	92.52
Quant-Noise [12]	8/8	24.8 M	6.27 G	93.66	99.74	80.61	96.70	72.12	89.75
FQ-ViT [10]	8/4	13.4 M	3.21 G	92.21	99.60	77.84	95.84	64.79	86.58
XNOR-Net [33]	1/1	4.8 M	3.21 G	75.17	98.28	57.61	82.79	48.45	73.04
BiBERT [34]	1/1	4.8 M	3.21 G	80.13	98.77	58.45	83.04	50.32	77.73
BiT [35]	1/1	4.8 M	3.21 G	80.40	98.78	60.31	85.98	51.01	77.99
BiViT [36]	1/1	4.8 M	3.21 G	82.28	98.85	61.08	85.50	50.59	78.84
BinaryViT (Baseline) [30]	1/1	5.0 M	3.27 G	84.35	99.04	63.84	86.42	53.33	78.22
BinaryFormer [16]	1/1	4.8 M	3.19 G	87.07	99.30	64.27	86.22	54.46	80.82
ACD-BiT (ours)	1/1	5.0 M	3.27 G	**89.72**	**99.67**	**65.49**	**88.92**	**58.76**	**81.61**

bolded numbers signify optimal results.

**Table 3:** Ablation experiments on distributed alignment (Da) and distillation correction (Dc). The evaluation metric is the accuracy of the first category (Top-1 Acc (%)) and the first five categories (Top-5 Acc (%)).

Modules		W/A	CIFAR10 (%)		CIFAR100 (%)		TinyImageNet (%)	
**Da**	**Dc**		**Top-1**	**Top-5**	**Top-1**	**Top-5**	**Top-1**	**Top-5**
-	-	1/1	84.35	99.04	63.84	86.42	52.14	75.98
√	-	1/1	87.61	99.27	64.62	87.81	54.13	76.76
-	√	1/1	86.21	99.06	64.30	87.75	52.83	76.13
√	√	1/1	**89.72**	**99.67**	**65.49**	**88.92**	**55.24**	**77.57**

bolded numbers signify optimal results.

## Data Availability

Data are contained within the article.
